# Eggs: Healthy or Risky? A Review of Evidence from High Quality Studies on Hen’s Eggs

**DOI:** 10.3390/nu15122657

**Published:** 2023-06-07

**Authors:** Madeleine Myers, Carrie Helen Stevenson Ruxton

**Affiliations:** 1 Non-Diet Nutrition, Chepstow NP16 5DN, UK; 2 Nutrition Communications, East Road, Cupar KY15 4HQ, UK

**Keywords:** eggs, cardiovascular, type 2 diabetes, obesity, protein, cholesterol, body composition, sustainability, allergy

## Abstract

Hen’s eggs (from *Gallus gallus domesticus*) provide choline, folate, vitamin D, iodine, B vitamins and high-quality protein and are no longer viewed by national bodies as a risk factor for hypercholesterolaemia and cardiovascular disease (CVD). Yet, questions remain about the benefits and risks of eating eggs regularly. This review evaluates recent high-quality evidence from randomised controlled trials (RCT) and meta-analyses of observational studies and considers new areas of interest, such as weight management, protein metabolism, allergy risk and sustainability. In several RCT, eggs increased muscle protein synthesis and lowered fat mass, which could support optimal body composition. Eggs within a meal improved satiety, which could translate into lower energy intakes, although more RCT are needed. In observational studies, higher egg consumption was associated with a null effect or a modest reduced risk of CVD. For type 2 diabetes (T2D) incidence and risk of CVD in people with T2D, there were inconsistencies between observational and RCT data, with the former noting positive associations and the latter seeing no effect of higher egg intake on markers of T2D and CVD. Sustainability metrics suggest that eggs have the lowest planetary impact amongst animal proteins. To lower allergy risk, earlier introduction of eggs into weaning diets is warranted. In conclusion, the balance of evidence points to eggs being a nutritious food suggesting there are broad health benefits from including eggs in the diet at intakes higher than that currently consumed by European populations.

## 1. Introduction

Hen’s eggs are widely consumed across all age groups within the global food system. However, there has been controversy on certain health topics, with public opinion sometimes lagging behind changing scientific evidence in recent decades. This has led to confusion about the benefits or harms of consuming eggs, particularly in relation to heart health [1]. Earlier concern that dietary cholesterol from eggs and other foods significantly raises plasma cholesterol levels and impacts heart disease risk has been replaced with the view that saturated fat intake has a greater impact [2]. In the last two decades, specific advice to limit egg consumption to around three per week was dropped by most health bodies in the UK and US, while limitations on dietary cholesterol intake in the UK were dropped in 2009 [1]. Although most countries removed dietary cholesterol restrictions from food recommendations earlier, the US retained theirs until 2015 [3].

Another debated aspect of egg consumption relates to food-borne disease that raw eggs may be contaminated with salmonella [4]. Significant improvements to egg production in the UK has resulted in updated advice to enable vulnerable groups to choose raw or lightly cooked eggs if they wish, as long as the eggs carry the British Lion mark [5]. Food-based dietary guidelines in many European countries now mention that eggs can replace meat and fish as a more sustainable protein source [6]. Some countries’ recommendations do not set a limit or give specific guidance on intakes but, where numbers of eggs are recommended, these vary from two to three per week in Finland and the Netherlands to seven eggs per week in Ireland and Bulgaria [6]. In the Flemish region of Belgium, the advice is to ‘eat no more than seven eggs per week otherwise you may increase your risk of diabetes’ [6]. These variations in recommendations highlight continued confusion about the links between specific intakes of eggs, health and risk of disease.

Eggs are eaten in meals, e.g., omelettes, or are used as a cooking ingredient in a wide range of composite foods, e.g., cakes. They are also one of the more affordable animal-protein sources [7]. If eggs are agreed to be nutritious and safe, advice to limit or avoid them could be counterproductive, especially in populations experiencing cost of living pressures. The purpose of this review is to consider evidence from studies relating to cardiovascular disease (CVD), metabolic health, weight management and body composition to weigh up the potential risks and benefits of regular egg consumption. The highest quality evidence was prioritised, including randomised controlled trials (RCT) and systematic reviews and meta-analyses (SRMA), and consideration was given to the nutritional composition and sustainability of eggs.

## 2. Materials & Methods

A search strategy was implemented in PubMed (covering January 2010–December 2022) focused on all SRMA of human studies, using the keyword ‘egg’ for all searches. This was combined with the following terms relevant for each health outcome of interest: ‘cardiovascular’, ‘cholesterol’, ‘stroke’, ‘coronary heart disease’, ‘diabetes’, ‘hyperglycaemia’, ‘glucose’, ‘metabolic syndrome’, ‘weight’, ‘obesity’, ‘satiety’, ‘appetite’, ‘sarcopenia’, ‘older adult’, ‘elderly’, ‘muscle’, ‘leucine’, ‘egg protein’, and ‘frailty’.

Studies were excluded if they reported health outcomes irrelevant to this review; for example, studies on oocytes or fertility, or involved consumption of the eggs of species other than *Gallus gallus domesticus*. Studies involved hen’s eggs produced by a variety of farming methods. Where no SRMA or RCT were available, the search strategy moved down the quality hierarchy to individual RCT, then finally to individual prospective cohort studies (PCS). SRMA were unavailable for the topics of weight management, satiety and body composition limiting the strength of the conclusions on these topics. Since only one SRMA was published for metabolic syndrome, the search strategy also included individual RCT.

## 3. Nutritional Benefits and Risks

### 3.1. Intakes in Europe

Europe is the second-largest producer of eggs in the world behind China, which far outpaces any other global area [8]. The average consumption of eggs in Europe was estimated to be 220–225 eggs per capita/year in 2021 [9]. For the United Kingdom, estimates were 198 eggs per capita/year or just under 4 eggs a week for the same year [10]. This is less than consumption in the USA and Canada, which reported intakes of 285 and 253 eggs/capita/year respectively in 2021. One of the top consuming countries in the world is Mexico where recent estimates are of 409 eggs per capita/year [9].

### 3.2. Overview of Egg Nutrition

Eggs are a moderate energy, nutrient-dense food providing 66 kcal, 6.4 g of protein and a wide range of micronutrients per medium egg, as shown in Table 1 [11]. A medium sized egg contains 4.6 g of total fat, with 1.7 g of this being monounsaturated fat. Alongside crustaceans and offal, eggs are rich in dietary cholesterol, providing 177 mg per medium egg. Cholesterol was viewed in the past as a negative nutrient, although risk assessment has evolved in line with newer evidence suggesting a lesser impact of dietary cholesterol on CVD risk versus saturated fat [12]. Egg yolk is one of the few naturally occurring food sources of vitamin D and the lipid matrix is believed to enhance bioavailability of yolk phytonutrients, such as lutein and zeaxanthin, although levels in the eggs depend on the hens’ diets and are lower in some countries, e.g., the UK [13].

According to the European Commission Nutrition and Health Claims Regulation [15], various claims can be made in relation to the nutrient content of eggs (Table 2). Eggs are a ‘source of’ pantothenic acid, phosphorus, vitamin A and folate. In addition, eggs are ‘high in’ protein, monounsaturated fatty acids, vitamin D, vitamin B12, biotin, riboflavin, selenium and iodine. For each of these nutrients, the relevant health claims listed in Table 2 could be applied to eggs in commercial communications, including labelling [16]. Although there is currently no Dietary Reference Value for choline in the UK, a health claim for choline can be applied if a food provides at least 82.5 mg/100 g. Eggs comfortably exceed this cut-off by providing 285 mg/100 g [17].

Potassium, calcium, iron, iodine, folate, vitamin D and fibre are highlighted by the European Food Safety Authority (EFSA) as nutrients of concern for the European population [18]. Of these, choline, vitamin D, folate and iodine are provided by eggs in clinically useful quantities.

### 3.3. Choline

Choline is an essential micronutrient for infant growth and development and is typically found in eggs and beef liver, with lower amounts in plant-based foods such as almonds and broccoli [19]. Recommendations have been set by some expert bodies; for example, the Average Intake (AI) set by the EFSA are 400 mg/day for adolescents and adults; and 480 mg/day during pregnancy. The values set by US health bodies are 425 mg for female adults; 550 mg for male adults; and 450 mg during pregnancy [20,21]. An analysis of choline intake in nine European countries found that the average intake was well below that of the American AI [22]. 

Choline intakes are also low in pregnancy, with only 8.5% of women meeting the American AI according to a nationwide survey [23]. There is some evidence that increasing choline intake during pregnancy above the AI may benefit infant cognitive development. Two studies found that daily choline supplementation (930 mg, roughly twice the AI) in the third trimester of pregnancy improved infant information processing speed at 4–13 months and improved sustained attention at 7 years compared with supplementing at the current AI of 480 mg/day [24,25]. Since eggs provide a rich source of choline, they can boost choline status in groups with higher requirements and typically low intakes. In an Australian study, eggs were the most significant contributor to choline intake in the diets of pregnant women [26]. The previously mentioned study by Wallace et al. found that egg consumers had significantly greater choline intakes compared with non-consumers (525 mg/day vs. 294 mg/day, *p* < 0.0001) [23]. The efficacy of whole eggs for raising plasma choline levels is confirmed by another RCT where participants given daily whole egg, but not a yolk-free egg substitute, had significantly higher plasma choline levels compared with baseline (*p* = 0.01) [27]. 

### 3.4. Vitamin D

Attaining an optimal vitamin D status can be challenging since few foods are natural sources and, in all countries above 37 degrees latitude, sun-induced vitamin D synthesis cannot occur during autumn and winter months. In the Northern Hemisphere and in certain populations—those with darker skin pigmentation, limited sun exposure and who cover their skin for religious or cultural reasons—dietary sources of vitamin D, including supplements, are critical [28]. An assessment of European dietary intakes suggests that mean vitamin D intake from food was just 2.7 μg in women and 3.3 μg in men, a stark contrast to the current UK recommendation of 10 μg per day [29,30] and the new Irish recommendation of 15 μg per day [31]. Since eggs are one of the few foods naturally high in vitamin D, they present an opportunity to help minimise the shortfall in vitamin D intake. A recent RCT in 51 Australian adults found that, after 12-weeks of either 2, 7 or 14 eggs/week during winter months, serum 25(OH)-vitamin D levels had significantly reduced only in the group consuming the lowest number of eggs [32]. This study suggests that consuming between 7–14 eggs/week may help to attenuate the typical seasonal drop in circulating 25(OH)-vitamin D.

### 3.5. Folate

Several populations are at risk of low folate intakes. Insufficient folate/folic acid in women of childbearing age means their offspring would be sub-optimally protected against neural tube defects. Data from the most recent National Diet and Nutrition Survey (NDNS) in the UK found that 89% of women of childbearing age had a red blood cell folate concentration below the threshold indicating increased risk for NTDs (748 nmol/L) [33]. A recent review of nutritional requirements for adults aged ≥ 65 years proposed that intakes of 400 μg were deemed more appropriate for this age group than the current UK recommendation of 200 μg [30,34]. This is due to the increased risk of deficiency in older people, which is associated with neurological damage and potentially increased risk of dementia [35]. A secondary analysis of the NDNS data found that ‘health conscious’ egg consumers (consuming > 3 eggs/week and low intake of red or processed meat) had significantly higher intakes of folate than those consuming high amounts of red and processed meats and no eggs [36]. In addition, a more recent analysis of the NDNS data by Gibson et al. found that female consumers of eggs had significantly higher intake of folate as well as protein and omega-3 fatty acids [37]. It is likely that health-conscious egg consumers also ate more fruit and vegetables which would have contributed to folate intakes. 

### 3.6. Iodine

Average European intake of iodine in females is estimated to be 127 μg, which is lower than the World Health Organisation (WHO) recommendation of 150 μg/day [29,38]. Iodine is an essential nutrient for normal foetal brain development and a low status may impact fertility and the risk of preeclampsia [39]. One prospective study suggested that long-term iodine intake from food sources may be more important for optimal behavioural outcomes in infants than iodine supplementation during pregnancy [40]. As eggs are high in iodine, they could provide a useful addition to the diet both prior to and during pregnancy to aid sufficient iodine intake.

### 3.7. Protein

Eggs are considered a source of high-quality protein since they contribute all nine essential amino acids. Objective measures of protein quality and digestibility give a high value for eggs (97%), which is similar to that applied to other high value animal protein sources such as milk and cheese (95%) and meat (94%) [41].

The previously mentioned review by Dorrington et al. suggests that older adults are likely to have higher protein requirements and advises age-specific recommendations [34]. High-quality protein consumed frequently across the day supports myoprotection in combination with appropriate exercise, which helps to prevent loss of skeletal muscle mass and function in older adults [42]. The current protein recommendation for adults, as proposed by EFSA, is 0.83 g/kg body weight daily, whereas the recommendation made in the aforementioned paper is for 1.2 g/kg body weight daily, equating to 84 g for a 70 kg person [43]. Two medium eggs provide 12.8 g of high-quality protein which would make an important contribution to protein recommendations. 

## 4. Results: Risk Assessment

### 4.1. Cardiovascular Health and Cholesterol

Table 3 summarises the findings of SRMA of RCT (*n* = 4) which considered links between egg consumption and cardiovascular health. SRMA of RCT provide the highest quality of evidence for examining health outcomes. The number of eggs consumed in the intervention arms ranged from 5 to 42 eggs/week and the control foods varied greatly, often resulting in high heterogeneity.

The SRMA by Wang et al. found that intakes of >4 eggs/week had no significant effect on blood pressure or blood lipids when compared with ≤4 eggs/week [45]. Participants were older adults consuming 7–21 eggs per week on average. Significant increases in low-density lipoprotein cholesterol (LDL-c) of a magnitude of +5.5–8.1 mg/dL were reported in three out of four studies in this SRMA [44,46,47]. Total cholesterol (TC) significantly increased in two studies by +5.6–9.1 mg/dL [44,47]. Since high-density lipoprotein cholesterol (HDL-c) also rose in two of the studies, this resulted in null findings for the lipid ratios [44,47]. Ratios between individual lipid markers are considered to be better predictors of CVD risk than individual markers [48].

The largest SRMA, which included 66 studies in 3185 participants, conducted a dose-response analysis for egg intake [47], finding a positive linear relationship between TC, HDL-c, TG and LDL-c/HDL-c ratio. However, a non-linear relationship was found for LDL-c and TC/HDL-c. This study found a smaller impact on blood lipids in studies more than 12 weeks long, suggesting adaptation over time. The SRMA by Rouhani et al. also found a positive linear relationship for HDL-c, but not for other blood lipids [44].

Table 4 summarises the SRMA of PCS (*n* = 15) which examined associations between egg consumption and CVD, coronary heart disease (CHD), stroke, coronary artery disease (CAD), ischaemic heart disease (IHD) and heart failure. Since CHD, CAD and IHD are considered to be the same condition, all are grouped under ‘CHD’ for the purposes of this review. One SRMA found that, when compared with no consumption, eating 6 eggs/week was associated with a 4% decreased risk of CVD events or mortality with similar findings found for 1–5 eggs/week [49]. Another SRMA in contrast reported a significant 19% increased risk of CVD when the highest and lowest egg consumers were compared [50]. The authors performed a dose-response analysis finding that, for each increment of 4 eggs/week, the overall CVD risk was 6% greater. A further SRMA also found that overall consumption of each additional egg/day was associated with a small but significant increase in risk of CVD of 4% [51]. However, the other three SRMA looking at risk of CVD found no significant effects of egg consumption [52,53,54] and studies on CVD mortality largely found no association between egg intake and risk of death from CVD [49,55,56,57,58]. The exception was the SRMA by Yang et al., which reported an overall 7% increased risk of CVD mortality with each increment of 1 egg/day [59].

Stroke risk was found to be non-significantly associated with egg consumption in most studies which examined this [49,52,53,54,60,62]. When comparing highest versus lowest egg intake, there was a 9–12% reduced risk of stroke [55,61]. Consuming up to 3.5 eggs per week was associated with a significant reduction in stroke risk, but this became non-significant at higher intakes. Tang et al., conducted a dose-response analysis, finding mixed results. At intakes of 1–4 eggs/week, there was a decreased risk of stroke which switched to an increased risk in those consuming 10+ eggs/week [62]. None of the studies found a significant association between egg intake and risk of stroke mortality [49,55,57,58,59].

The majority of studies examining CHD risk found no associations with egg intake [52,53,55,60,61,63]. Godos et al. reported that eating up to 2 eggs/week was associated with a 4% decreased risk of CHD incidence/mortality with similar findings at higher intakes [49]. Another SRMA found a significant 11% reduced risk when comparing higher consumers (>1 egg/day) to lower (≤1 egg/day) [54]. No associations between egg intake and CHD mortality risk were found [55,57,58]. The one SRMA to look at heart failure risk found that intakes of 7 eggs/week was associated with an increased risk of 15%, rising to 23% for 9 eggs/week, when compared with no consumption [49].

Five SRMA looked at CVD risk in populations with diabetes but three studies [50,52,60] did not state the type. It is inappropriate to combine data on type 1 and type 2 diabetes (T2D) as they have different origins [64]. Li et al. found that people with non-specific ‘diabetes’ eating the highest number of eggs had an 83% increased risk of CVD compared with the lowest consumers [50]. In addition, for every 4 egg/week increment, the relative risk was 40% greater. Similar results were seen in another SRMA of ‘diabetes’ where CVD risk was 69% greater for highest versus lowest egg consumers [52]. The SRMA by Drouin-Chartier et al., in those with T2D, found a 40% increased risk for highest egg consumers and an overall 25% increased risk for each additional egg per day [53]. A further SRMA looking at CHD found a 54% increased risk in highest versus lowest consumers [60]. However, another SRMA examining risk of CHD found no significant association, even at intakes of 7+ eggs per week [63].

In summary, evidence from RCT suggests that eggs tend to have overall small effects on blood cholesterol levels. Evidence from observational studies is conflicting depending on whether the baseline population is healthy (in which case eggs have a modest beneficial association or no association with CVD risk) or has pre-existing diabetes (in which case eggs are associated with greater CVD risk at higher intakes). 

### 4.2. Metabolic Health

Six SRMA of PCS were found for this topic (Table 5) which all had risk of T2D as the primary outcome except for Li et al. [50] which did not specify the type of diabetes. In the three SRMA which compared highest and lowest eggs consumers, the highest had a 68%, 42% or 9% increased risk of developing diabetes, respectively [50,52,65]. Li et al. also conducted a dose-response analysis, finding a 29% greater risk of developing non-specific diabetes for each additional serving of 4 eggs/week [50]. Two other SRMA which conducted dose-response analyses found that, overall, each egg per day was associated with a 7–13% increased risk of T2D [66,67]. In a further dose-response analysis, Djousse et al. reported that T2D risk was elevated by 7% but only for >4 eggs/week [65]. Another SRMA found no association with 1 egg/week, but intakes of ≥2 eggs/week were associated with an 11–27% increased risk of T2D compared with non-consumption [63].

Assuming from the observational data that high egg consumption increases the risk of T2D, one would expect to see a similar signal for eggs and metabolic syndrome (MetS). However, this is not the case. The only SRMA found for this topic summarised 19 PCS in 331,667 young to middle aged participants, finding that higher egg consumption was associated with a significant 8% reduction in risk of MetS [68]. The authors noted that 17 of these studies were conducted in Asia and may not be representative of other global populations.

Six relevant publications reporting RCT findings were also found (Table 6). Three examined higher egg intake (3 eggs/day) in combination with a moderate carbohydrate-restricted diet for 12 weeks in 37 participants with MetS and found significantly beneficial effects on metabolic health for higher egg consumption compared with control groups. These effects included higher HDL-c, larger HDL-c particles, lower levels of very low-density lipoproteins (VLDL), insulin, HOMA-IR and LCAT activity, no change to LDL-c or TC, and improved status of antioxidants and carotenoids [69,70,71]. Such changes conflict with the findings from observational studies and do not fit with the theory that eggs cause T2D.

Two publications reported the results of a crossover RCT where 24 participants with MetS consumed either 2 eggs/day versus yolk-free egg substitute plus 70 g spinach/day for 4 weeks [72,73]. As well as reporting similar metabolic changes to other RCT (higher HDL-c, larger HDL-c particles, higher carotenoids, improved antioxidant status), there was a modest, statistically significant reduction in weight and body mass index. Since the control food in these RCT was a yolk-free egg substitute, the beneficial effects could be due to nutrients or bioactive compounds in the egg yolk.

**Table 6:** Intervention studies on eggs and metabolic risk markers.

Author, Year	Study Design	Intervention	Number of Participants	Outcomes	Results
Andersen, 2013 [69]	SBRCT, parallel	3 eggs/day or yolk-free egg substitute + moderately carbohydrate-restricted diet for 12-weeks	37 adults with MetS (30–70 y)	Blood lipids	Egg group had significantly greater increase in HDL-c vs. egg substitute group No significant change to LDL-c or TC.
Blesso, 2013 [70]	SBRCT, parallel	3 eggs/day or yolk-free substitute + moderately carbohydrate-restricted diet for 12-weeks	37 adults with MetS (51.9 ± 7.7 y)	Carotenoid status	Egg group had significantly increased plasma lutein and zeaxanthin vs. egg-substitute group. Significant lipoprotein enrichment with lutein and zeaxanthin in egg group only.
Blesso, 2013 [71]	SBRCT, parallel	3 eggs/day or yolk-free substitute + moderately carbohydrate-restricted diet for 12-weeks	37 adults with MetS (51.9 ± 7.7 y)	Blood lipids, insulin resistance	Egg group had significantly greater increases in HDL-c, large HDL-c particles, LCAT activity and HDL-c and LDL-c diameters vs. egg-substitute group Egg group had significantly reduced plasma insulin, HOMA-IR and VLDL-c vs. egg-substitute group.
Thomas, 2022 [73]	RCT, crossover	2 eggs/day with 70 g spinach or yolk-free egg substitute with 70 g spinach + meat-free diet for 4 weeks	24 adults with MetS (49.3 ± 8 y)	Oxidative stress, inflammation	Egg group had significantly lower plasma malondialdehyde compared with egg substitute group. No significant differences in other biomarkers. MetS characteristics reversed in 11 participants during the egg diet and 7 participants during the egg substitute diet.
Thomas, 2022 [72]	RCT, crossover	2 eggs/day with 70 g spinach or yolk-free egg substitute with 70 g spinach + meat-free diet for 4 weeks	24 adults with MetS (49.3 ± 8 y)	Inflammatory markers, blood lipids	Egg group had significant reduction in weight and BMI compared with egg substitute diet. Egg group had significant increase in HDL-c, large HDL-c particles and choline compared with baseline Plasma zeaxanthin rose significantly during egg diet compared with egg substitute diet and with baseline.
Thomas, 2022 [74]	RCT, crossover	3 eggs/day or choline supplement for 4 weeks	23 adults with MetS (35–70 y)	Plasma TMAO, carotenoid status, gut microbiome	Significant increases in plasma choline seen in both interventions No change to plasma TMAO or gut microbiome during either intervention. Plasma lutein and zeaxanthin increased during egg intervention relative to baseline and choline treatment.

Key: BMI, body mass index; HDL-c, high-density lipoprotein cholesterol; HOMA-IR, Homeostatic Model Assessment for Insulin Resistance; LCAT, Lecithin-cholesterol acyltransferase enzyme; LDL-c, low-density lipoprotein cholesterol; MetS, metabolic syndrome; RCT, randomised controlled trial; SBRCT, single blind randomised controlled trial; TC, total cholesterol; TMAO, trimethylamine *N*-oxide; VLDL-c, very-low-density lipoprotein cholesterol; y, years.

In the final RCT in 23 participants with MetS, 3 eggs/day or a choline supplement was given for 4 weeks [74]. Plasma antioxidants significantly increased during the egg intervention compared with baseline and choline. Plasma choline significantly increased during both interventions but there were no concurrent increases in plasma trimethylamine *N*-Oxide (TMAO) during either treatment. TMAO, a marker of chronic disease risk, has been shown in other studies to correlate with choline intakes [75]. Significant changes to the gut microbiome were also not seen in this study.

In summary, observational and RCT findings directly conflict on the issue of whether eating eggs raises or lowers the risk of T2D. Hence, it is implausible that eggs play a causal role in the development of T2D and there is evidence that nutrients or bioactives in the yolk may have a positive impact on metabolic markers.

## 5. Results: Benefits Assessment

### 5.1. Weight Management and Satiety

Seven RCTs examined associations between egg intake and markers of weight management with most reporting effects on energy intake or satiety (Table 7). Only one measured weight change after providing 128 participants with T2D or MetS an energy-restricted high or low egg diet for 3 months [76]. Although weight was lost during both intervention periods, there was no difference in rates of weight loss between groups. In contrast to the previous section which found improvements to metabolic markers after egg consumption, no changes to glycaemic, lipid, oxidative or inflammatory markers were found in populations with pre-existing T2D. 

Two RCT looked at child and adolescent populations, finding no effects on appetite or satiety after egg consumption. However, Liu et al. [80] reported higher levels of PYY—an appetite-suppressing hormone—three hours after a breakfast with eggs compared with bagels. Kral et al. found a lower energy intake at lunch following an egg breakfast versus a cereal or oatmeal breakfast [83]. 

In adults, three RCT reported that an egg breakfast led to significantly lower energy intakes compared with an energy-matched high carbohydrate breakfast [77,79,82]. Two of these studies additionally found altered signals for appetite hormones [77] or satiety/desire to eat [79]. While Pombo-Rodrigues et al. [78] found no differences in energy intake after a lunch of either an omelette, jacket potato or chicken sandwich, participants reported feeling fuller and less inclined to eat after the omelette versus the other meals. 

It is unclear whether these effects on satiety/energy intake relate to the amino acid composition of eggs or another nutritional factor. A 7-day study [84] with protein-matched breakfasts found no differences in energy intake, hormone levels or reported satiety when comparing eggs with cereal. In contrast, a 3-month study [81] found reduced hunger and increased satiety in people with T2D after protein-matched breakfasts which were high or low in eggs. The longer duration of this study may explain the differing results.

In summary, evidence from RCT indicates that eggs are a satiating food which may reduce energy intake at subsequent meals, helping to support weight management.

### 5.2. Myoprotection in Adults

Eight RCT had relevance to links between egg intake and muscle mass/strength or protection against frailty/muscle loss in adults (Table 8). Looking first at studies on body composition and strength, three compared whole egg versus egg white for changes in body composition, muscle protein synthesis and strength [85,86,87]. Two of these reported findings which implied that whole eggs have an advantage over egg whites in terms of boosting muscle protein synthesis [85] or improving markers of strength and reducing body fat [86]. It is known that myofibrillar protein synthesis must exceed protein breakdown for hypertrophy of muscles [88]. However, another RCT with a similar methodology found that both types of egg led to significant beneficial changes in body composition and skeletal muscle regulatory markers [87]. 

A longer term 8-week RCT compared egg white and carbohydrate supplementation in young female athletes. Beneficial changes in body composition and strength were seen in both groups, yet only the egg group increased protein metabolites [89]. Taken together, these studies indicate that both whole egg and egg white improve muscle protein synthesis and body composition—probably since their protein content is similar—but whole egg might have the edge in relation to fitness and myoprotection.

Protein quality may be important with differences between animal versus plant proteins. An RCT in 56 young adults revealed that animal proteins resulted in greater whole-body anabolic response than plant proteins [90]. In addition, eggs suppressed protein breakdown to a greater extent than mixed nuts. Another RCT found that an egg breakfast led to a significantly higher net protein balance and reduced protein breakdown compared with a cereal breakfast [91].

Two RCT were performed in older adults. The first gave elderly women egg white protein or a carbohydrate supplement for 6 months, finding no significant changes in measures of body composition or physical function [92]. However, hand grip strength and strength increased significantly in the egg group and more individuals met protein requirements. The authors suggested that limited recruitment of the target population and the high attrition rate could explain why other significant diet differences were not observed. A second RCT in older adults found that consumption of both a high protein egg-containing diet or a low protein egg-free diet for 12 weeks reduced body weight and body fat but only those on the high protein diet maintained lean mass while this declined on the low protein diet [93]. This result supports the view that a high protein diet is needed to preserve lean body mass during planned weight loss.

**Table 8:** Intervention studies on eggs and markers of myoprotection.

Author, Year.	Study Design	Intervention	Number of Participants	Outcomes	Results
Hida, 2012 [89]	DBRCT, parallel	Egg white protein vs. carbohydrate supplement for 8-weeks	30 female athletes (18–22 y)	Exercise performance and body composition	Serum urea increased in egg group. No difference between diet groups for change in muscle mass, strength tests or body fat.
Van Vliet, 2017 [85]	RCT, crossover	Whole egg vs. egg white (protein matched), acute	10 men (21 ± 1 y)	Post-exercise muscle protein synthesis	Protein-derived leucine appeared more rapidly in plasma following egg white. Whole egg significantly increased post-exercise myofibrillar protein synthetic response vs. egg white.
Kim, 2017 [91]	RCT, crossover, acute	Egg breakfast vs. cereal breakfast (protein matched), acute	12 adults (57–74 y)	Net protein balance (anabolic response)	Protein breakdown significantly lower and post-meal net protein balance significantly higher after eggs vs. cereal.
Wright, 2018 [93]	RCT, parallel	High protein diet (3 eggs/day) vs. normal protein diet (no eggs) for 12 weeks	22 adults (50–80 y)	Muscle and body composition	High protein egg diet significantly reduced subcutaneous fat to muscle volume in mid-calf. Low protein egg-free diet significantly reduced lean mass and trunk mass. No differences in body weight.
Bagheri, 2020 [86]	SBRCT, parallel	Whole egg vs. egg white diet (protein matched) + resistance training for 12 weeks	30 men (24.6 ± 2.7 y)	Body composition, knee extensor muscle mass, muscular strength, anaerobic power, hormonal response	Whole egg diet significantly reduced percent body fat (−2.7%) and increased hand and quadriceps strength and serum testosterone vs. egg white diet.
Bagheri, 2020 [87]	SBRCT, parallel	Whole egg vs. egg white (protein matched) + resistance training for 12 weeks	30 men (24.6 ± 2.7 y)	Body composition, skeletal muscle regulatory markers	No significant differences between groups for body composition or muscle regulatory markers. Similar increases in body weight and muscle mass and reductions in body fat for both diet/exercise groups.
Park, 2021 [90]	SBRCT, parallel, acute	Beef vs. pork vs. eggs vs. kidney beans vs. mixed nuts vs. peanut butter vs. tofu	56 adults (18–40 y)	Net protein balance (anabolic response)	Whole-body net protein balance significantly greater following animal protein diets vs. plant protein diets. Compared with high mixed nuts, high pork and eggs suppressed protein breakdown to a greater extent.
Ullevig, 2022 [92]	DBRCT, parallel	Egg white protein supplement vs. carbohydrate supplement for 6-months	29 females (73.6 ± 8.3 y)	Body composition, strength and physical function	Hand grip strength and number of arm curls significantly increased from baseline in egg group only. No significant differences in body composition between groups.

Key: DBRCT, double blind randomised controlled trial; RCT, randomised controlled trial; SBRCT, single blind randomised controlled trial; y, years.

In summary, the available evidence in adults suggests that egg intake can beneficially affect protein metabolism and help to conserve lean mass. While the high value protein in eggs would be expected to contribute to myoprotection, there is some suggestion that the nutrients found in yolks could also play a role. 

## 6. Other Dietary Aspects Relating to Eggs

This section examines other considerations when determining the benefit and risks of regular egg consumption and is separate from the structured literature search undertaken for Section 4 and Section 5 as it includes expert opinion and official publications. Here, we consider sustainability, food safety and allergy, which are, nevertheless, important in modern discussions of public health nutrition.

### 6.1. Sustainability

Food production and consumption are exceeding planetary boundaries and contributing to climate change and loss of biodiversity. Hence, diets need to shift towards climate friendly options, but this demands a clear understanding of the evidence base upon which such diets should be based. Misconceptions are common, particularly around eggs and their place in a heathy sustainable diet. Eggs are often categorised with other animal proteins such as beef, lamb, poultry and dairy when reporting on the environmental impact of foods, but the environmental impact of egg production is considerably lower than these alternatives.

Research from several scientific reports suggests that eggs are responsible for less carbon, land and water use than other animal protein, particularly beef. The 2011 report by the World Wildlife Fund demonstrated that animal proteins are responsible for 57% of greenhouse gas emissions (GHGEs) but, of this, eggs were responsible for only 1.9% of GHGEs [94]. Data from the English National Food Strategy revealed that producing 100 g of protein from eggs creates 3.8 kg of CO_2_ equivalent on average, compared with 25 kg for beef [95,96]. Similarly, the British Dietetic Association has reported that, per 100 g of protein produced, eggs produce less GHGEs than beef (4.2 kg versus 50 kg of CO_2_, respectively) and use less land (5.7 m^2^ versus 164 m^2^ respectively) [97]. The same report concluded that eggs are responsible for only 1.8% of dietary GHGEs, compared with 24.2% for beef. The Eat Lancet report produced similar conclusions for the environmental impact of eggs relating to GHGEs, land and energy use and the potential for acidification and eutrophication [98]. Other recent reports and a meta-analysis have published similar findings [99,100,101]. On several environmental measures, eggs also differ from plant-based foods; for example, egg production uses much less water than almonds.

When considering sustainable diets, the large focus on GHGEs may cause land use—another important factor—to be overlooked. Of importance are data suggesting that diets containing plants, eggs and fish use the same amount of land as a vegan diet [94]. Hence, a vegan diet is not automatically more sustainable than a lacto-ovo-vegetarian diet, particularly if ultra processed plant-based meats and dairy products are used to replace animal foods [102] and can often be less nutritionally balanced [103]. Diets are shifting in the UK, but only about 3–4% of people consume vegan diets while 13% follow a flexitarian diet which is based on plants with limited animal protein [104].

### 6.2. Allergy Risk

Food allergies affect around 3–8% of children and 1–3% of adults. Egg allergy, as with most other food allergies in children, tends to be outgrown before adulthood [105]. Early advice given to parents was to delay the introduction of potentially allergenic foods (including wheat, peanuts, cow’s milk and eggs) during weaning. However, this has now been found to be counterproductive, and it is the current view that potentially allergenic foods should be introduced when weaning commences at around 6 months [106,107]. Indeed, contrary to previous beliefs, there is growing evidence that early introduction of potentially allergenic foods may reduce the risk of allergy in babies and children [108,109].

In 2018, a joint paper from the UK Scientific Advisory Committee on Nutrition and the Committee on Toxicity of Chemicals in Food, Consumer Products and the Environment (COT) was released with guidance that eggs need not be differentiated from other solid foods during weaning and can be introduced from 6 months [110]. The guidance for babies at higher risk of food allergy (i.e., those with eczema or other known food allergies) is to introduce cooked eggs into the diet from around 4 months of age and maintain intake [111,112]. 

### 6.3. Food-Borne Disease Risk

Beginning in the 1980s, human cases of the food-borne illness caused by salmonella steadily rose and were determined to be caused by consumption of eggs from chickens infected with pathogenic *Salmonella* serovars [4]. In 1998, the Lion Code of Practice was introduced in the UK to help tackle this and ensure increased hygiene control, traceability, testing of flocks and vaccination against *Salmonella enteritidis* and *Salmonella typhimurium*. Just two years after implementation of the Lion Code of Practice, human cases of salmonella dramatically reduced [5]. Countries which have not implemented such safeguards have rates of *Salmonella* in eggs many times higher than those found in the UK; figures in 2012 reported this was just 0.07% [113]. More than 90% of all UK eggs are produced to the Lion Code of Practice and bear the British Lion mark [114].

In 2017, the Food Standards Agency updated their guidance to expand on the groups that could safely consume raw or lightly cooked eggs, provided they bear the Lion Quality mark, including infants, children, pregnant women and the elderly [115]. This change was prompted in part by a report from the Advisory Committee on the Microbiological Safety of Food which showed that salmonella contamination of UK eggs has plummeted in recent decades [5]. 

## 7. Discussion of Benefits vs. Risks

In bringing together SRMA and other high-quality evidence, this review adds to the existing literature by highlighting the overall trend in benefits versus risks of eggs consumption, which is difficult to see when one health topic is considered. The overview also revealed discord between the findings of observational and intervention studies in relation to cardiometabolic risk, a phenomenon that has been noted by several authors [116,117]. For general populations, SRMA of PCS found neutral or beneficial effects of moderate egg consumption on CVD mortality and risk. Some SRMA of RCT found modest increases in serum lipid levels which may be due to the high intakes of eggs given during these short-to-medium term interventions which do not reflect mean habitual intakes. However, HDL-c levels typically increased during these studies which can rebalance lipoprotein profile and, in terms of disease risk, may mitigate some of the negative effects of higher TC or LDL-c. There is some evidence that individuals respond differently to dietary cholesterol depending on whether they are categorised as having high rates of cholesterol synthesis (low absorbers) or high rates of cholesterol absorption (low synthesisers) [13].

This discord was more evident when examining links between egg consumption and T2D. SRMA of PCS reported an increased risk of developing T2D when participants ate eggs more regularly and an increased risk of CVD in higher egg consumers with pre-existing T2D. ‘Higher’ in these studies ranged from 2 to 7+ eggs per week. In contrast, RCT in participants with MetS—arguably at higher risk of T2D and CVD than the general population—reported neutral or positive effects on disease markers including insulin resistance and serum lipids. In these RCT, 2–3 eggs per day were consumed, which is in excess of the levels of intake deemed to be ‘high’ in observational studies as well as habitual intakes in Western nations. What could be the reasons for this lack of alignment between different types of studies?

A major limitation of observational studies is that they are not designed to determine causality; hence, confounding is a problem, particularly since eggs are generally not eaten in isolation and represent only a tiny proportion of daily energy intake (2% in the UK NDNS on average) [33]. Dietary confounders, such as higher intakes of processed meats, saturated and trans fatty acids, and food energy, as well as lower fruit and vegetable intakes, have been proposed as explanations for the positive associations observed between higher egg consumption and T2D or CVD in some PCS. In national surveys, higher egg intake can be a marker of higher body mass index and less healthy dietary patterns characterised by fatty, processed meats and fried foods typically seen in traditional British or US breakfasts [13,36]. This is supported by a risk apportionment study based on US adult data which found that consuming one egg a day accounted for <1% of CHD risk compared with 40% represented by all modifiable lifestyle risk factors [118]. Hence, other factors are more important than eggs for CHD risk.

Mention should be made of the mechanisms proposed to explain statistically significant associations between high consumption of eggs and T2D incidence, or risk of CVD in populations with T2D. These include hypotheses that cholesterol in eggs increases serum LDL-c; choline in eggs raises trimethylamine *N*-oxide (a metabolite produced by gut bacteria which has been associated with T2D and CVD risk); and that eggs may impact oxidative or inflammatory markers. None are supported by high-quality clinical evidence. RCT which provided 2–6 eggs daily in healthy [75,119,120] and at risk [27] groups of participants confirmed that eggs do not raise TMAO levels. Similarly, RCT based on daily egg intake found no adverse effects on markers of inflammation or oxidative stress [72,73,120]. Indeed, adding whole eggs or egg white to a glucose dietary challenge in prediabetic men attenuated post-prandial oxidative stress [121].

The hypothesis that cholesterol in eggs influences serum lipids in people with T2D, hence increasing their risk of CVD, is incongruous with the observation that T2D is associated with reduced cholesterol absorption in the gut and increased synthesis in the liver [122]. In a double-blind, RCT trial during which healthy subjects consumed zero, two and four egg yolks per day for 4 weeks, the LDL-c rise with egg feeding was attenuated in insulin resistant participants, regardless of obesity status [123]. The authors proposed that this was due to diminished cholesterol absorption. Hence, in view of this, serum cholesterol in people with T2D is likely to be less sensitive to dietary cholesterol from foods such as eggs [13]. Indeed, in two RCT on participants with T2D fed high egg diets, levels of LDL-c were unchanged after 12 weeks and other CVD markers showed improvements, indicating that egg consumption was beneficial, not harmful, in these groups [81,124]. These findings are supported by a systematic review of RCT which concluded that eating 6–12 eggs/week did not significantly affect TC, LDL-c, TG, fasting glucose, insulin or *C*-reactive protein in people with prediabetes or T2D, while HDL-c increased in several studies [125]. 

In relation to body composition, eggs appear to increase overall protein intake which could beneficially affect protein metabolism and conserve lean mass. Protein-rich diets prevent muscle loss and may lower the risk of frailty if combined with appropriate exercise [42]. Additionally, current research suggests that rapidly digested protein with high proportions of essential amino acids and adequate leucine (700–3000 mg) are most effective in stimulating muscle protein synthesis [126]. Eggs contain a source of leucine—approximately 15 g of egg white contains 1341 mg leucine—making it an option for meeting the requirements for maximal stimulation of muscle protein synthesis [89]. Hence, eggs could be a familiar and low-cost way to deliver high-quality protein and micronutrients into the diets of older people [127]. Eggs can be incorporated into many different meals that are likely to be widely accepted, especially since they provide no barrier for people with poor dentition. As most studies on this topic at present are in young or middle-aged adults, there is a need for future RCT in older populations which could look at protein synthesis, frailty and markers of sarcopenia.

Most studies which examined subjective measures of satiety or energy intake, mostly in the context of eating eggs at breakfast, found beneficial effects on satiety and a reduction in subsequent energy intake. These effects could have a positive impact on weight management by helping to prevent body weight gain or supporting weight loss as part of energy restricted diets. The finding in one study that including eggs in a diet helped to maintain lean body mass, probably due to their high protein content, is relevant for weight management, since lean body mass is metabolically active [93]. In the four studies that found no significant differences for satiety or energy intake, two were in children/adolescents and one involved dietary energy restriction [80,83,84]. Further studies should examine the effects of including eggs in weight management diets to determine the long-term weight and health consequences of the satiating effects of eggs. There is also a need for future interventions to measure the full range of outcomes including reported satiety, energy intake, hormone levels and weight change, as current data are patchy.

Despite the benefits indicated by several studies, average intakes of eggs remain low; in one UK survey, 40% of individuals did not consume eggs during the study period [37]. This highlights an opportunity for more people to include eggs in their diets on a regular basis. Incorporating eggs into the diets of children, adults and older people would improve intakes of specific nutrients of concern: in particular, folate, iodine, choline and vitamin D. This could be important in populations at risk of nutrient inadequacies and at important life stages such as infancy, old age and child-bearing years in women. In a prospective study of 2690 infants aged 6–24 months, egg consumption was associated with significantly greater choline intakes and greater recumbent length [128]. No specific recommendations could be found for an optimal intake of eggs. However, from a nutritional perspective, 7–14 eggs/week within a varied and balanced diet could be beneficial for most of the population in terms of increasing nutrient density and providing high-quality protein which can protect lean body mass and improve the satiating quality of meals.

Turning to sustainability, which is an important consideration due to climate concerns, recent research and scientific reports demonstrate a remarkable consensus that eggs have a lower environmental impact than other animal proteins. It is therefore unjustifiable to categorise eggs with meat and dairy when providing advice to the public on climate-friendly diets. Compared with other animal proteins, eggs combine high protein quality with a relatively lower impact on GHGEs. Eggs can, therefore, contribute to balancing reductions in environmental impact whilst supporting optimal nutrition and would be the best animal-based protein to recommend to those following plant-based and flexitarian diets.

In terms of allergy risk, the previous advice was to delay introduction of eggs in weaning. More recently, two landmark studies concluded that early introduction of allergenic foods may lower risk of food allergy in children; hence, the advice to parents has been updated [106,107]. Yet, despite this, egg intake in infants remains low. A recent UK survey estimated that only 54% of 6–8-month-olds have ever been offered eggs [129]. It is important to disseminate this recent information about the safety of eggs in weaning diets which could help to reduce the risk of allergy development and provide babies with a sustainable, high protein, nutrient-rich weaning food. The change of food safety advice to recommend that eggs with the British Lion mark can be offered raw or lightly cooked to vulnerable groups, including babies, pregnant women and elderly people is also significant as it provides greater cooking and serving options for eggs in the diet.

Limitations of the current study include not taking a fully systematic approach to literature searching, meaning that relevant papers may have been missed. In addition, only one database was used for the search. A strength was the preference for SRMA, which provide the highest quality of evidence.

## 8. Conclusions

Eggs are highly nutritious, accessible and affordable. Evidence from high-quality studies suggests they have a positive or neutral impact on health markers and do not pose a risk when eaten regularly as part of a balanced diet. Current egg consumption in the UK is low, providing scope for more families and individuals to eat eggs more often. For groups with high nutrient requirements, such as the elderly, infants, children, pregnant women and athletes, eggs represent a high-quality source of protein that provides key micronutrients, such as vitamin D, iodine, folate and choline, which are often below recommended levels in habitual diets. For the general population, eggs are emerging as one of the most sustainable options for a high-quality animal protein source which will be of benefit as more people switch towards flexitarian or vegetarian diets. In addition, given their impact on satiety and myoprotection, regular consumption of eggs could help support optimal weight management, an important consideration given the burden of obesity and related non-communicable diseases in Western countries. Finally, to answer the question posed in the title of this review, the balance of evidence points towards eggs being nutritious, healthy and sustainable, rather than risky.

## Figures and Tables

**Table 1:** Nutritional composition of UK hens’ eggs.

Nutrient	Per 100 g Whole Raw Egg	Per Medium Egg ^a^ 58 g	% Recommendation ^b^ Per Serving (2 Eggs)
Energy kcal	131	66	7
Protein g	12.6	6.4	26
Carbohydrate g	Tr	Tr	Tr
Fat g	9	4.6	13
Saturated fat g	2.52	1.3	13
Monounsaturated fat g	3.44	1.7	-
Cholesterol mg	350	177	-
Vitamin A μg	126	64	16
Vitamin D μg	3.2	1.6	64
Riboflavin mg	0.5	0.25	10
Folate μg	47	24	24
Vitamin B12 μg	2.7	1.4	112
Choline mg	285	144	72
Biotin μg	19.5	9.9	40
Phosphorus mg	179	91	26
Iron mg	1.72	0.9	13
Zinc mg	1.1	0.6	12
Iodine μg	50	25	33
Selenium μg	23	12	44
Pantothenic acid mg	1.35	0.7	12

^a^ Refers to edible portion of an average medium egg (58 g) [11]; ^b^ Refers to % EU Reference Intake for macronutrients and % Nutrient Reference Value for micronutrients [14].

**Table 2:** Nutrition and health claims permitted for hens’ eggs in Europe and UK.

Nutrient	Cut Offs for ‘Source of’ and ‘High in’ Claims	Content in 100 g of Egg (Edible Portion)	Permitted Nutrition Claim for Eggs	Health Areas Where Authorised Health Claims Apply
Protein	Source of = 12% of energy provided by protein; High in = 20% of energy provided by protein	38.5% of energy provided by protein	High in protein	Growth and maintenance of muscle mass, maintenance of bones.
Vitamin D	Source of = 15% of RDA; High in = 30% of RDA	64%	High in vitamin D	Normal bones and teeth, absorption and utilisation of calcium and phosphorus; normal blood calcium levels, immune function.
Monounsaturated fatty acids (MUFA)	High in = provides >45% of total fatty acid content and >20% of energy value	49% of total fatty acid content from MUFA; 24% of energy from MUFA	High in MUFA	None authorised
Vitamin B12	Source of = 15% of RDA; High in = 30% of RDA	108%	High in vitamin B12	Red blood cell formation, energy metabolism, immune function, nervous system, psychological function, homocysteine metabolism, reduction in tiredness and fatigue.
Riboflavin	Source of = 15% of RDA; High in = 30% of RDA	35.7%	High in riboflavin	Energy metabolism, iron metabolism, vision, normal skin and mucous membranes, red blood cells, protection of cells from oxidative stress, nervous system, reduction in tiredness and fatigue.
Folate	Source of = 15% of RDA; High in = 30% of RDA	23.5%	Source of folate	Psychological function, blood formation, homocysteine and amino acid metabolism, immune function, maternal tissue growth during pregnancy, reduction in tiredness and fatigue.
Vitamin A	Source of = 15% of RDA; High in = 30% of RDA	15.7%	Source of vitamin A	Immune function, normal skin and mucous membranes, vision, iron metabolism.
Phosphorus	Source of = 15% of RDA; High in = 30% of RDA	25.5%	Source of phosphorus	Normal function of cell membranes, energy metabolism, normal bones and teeth.
Selenium	Source of = 15% of RDA; High in = 30% of RDA	41.8%	High in selenium	Protection of cells from oxidative stress, immune function, normal thyroid function, hair and nails, spermatogenesis.
Biotin	Source of = 15% of RDA; High in = 30% of RDA	39%	High in biotin	Psychological function, normal skin, hair and mucous membranes, nervous system.
Pantothenic acid	Source of = 15% of RDA; High in = 30% of RDA	22.5%	Source of pantothenic acid	Synthesis of steroid hormones, vitamin D and neurotransmitters, energy metabolism, mental performance, reduction in tiredness and fatigue.
Iodine	Source of = 15% of RDA; High in = 30% of RDA	33.3%	High in iodine	Normal thyroid gland function, production of thyroid hormones, energy metabolism, normal skin, cognitive function, nervous system.
Choline	Health claim allowed if 82.5 mg/100 g food	285 mg	None authorised	Normal metabolism of fat and homocysteine, maintenance of liver function.

**Table 3:** Intervention studies on eggs and cardiovascular risk markers.

Author, Year	Study Design	Number of Studies (Number of Participants)	Outcomes	Results
Rouhani, 2017 [44]	SRMA of RCTs	28 (1734)	Blood lipids	Egg consumption significant increased TC, LDL-c and HDL-c vs. controls. No significant effect on TC/HDL-c ratio, LDL-c/HDL-c ratio or TG.
Wang, 2019 [45]	SRMA of RCTs	9 (412)	Blood pressure, blood lipids	>4 eggs/week had no significant effect on blood pressure or blood lipids vs. ≤4 eggs/week.
Li, 2020 [46]	SRMA of RCTs	17 (not stated)	Blood lipids	Greater egg consumption significantly increased LDL-c/HDL-c ratio and LDL-c cholesterol vs. controls, particularly in studies with longer duration. No significant effect on HDL-c levels.
Khalighi Sikaroudi, 2020 [47]	SRMA of RCTs	66 (3185)	Blood lipids	Egg consumption significantly increased TC, LDL-c, HDL-c, TC/HDL-c ratio and serum apoB100. No significant effect on serum apoA1, TG, VLDL-c or LDL-c/HDL-c ratio.

Key: apo, apoprotein; HDL-c, high-density lipoprotein cholesterol; LDL-c, low-density lipoprotein cholesterol; RCT, randomised controlled trial; SRMA, systematic review and meta-analysis; TC, total cholesterol; TG, triglycerides; VLDL-c, very-low-density lipoprotein cholesterol; vs, versus.

**Table 4:** Observational studies on eggs and cardiovascular risk.

Author, Year	Study Design	Number of Studies (Number of Participants)	Outcomes	Significant Differences in Relative Risk between Highest and Lowest Egg Consumers
Li, 2013 [50]	SRMA of PCS	12 (226,784)	CVD	High consumers had 19% increased risk of CVD incidence (83% in those with unspecified diabetes). DR analysis: 6% greater risk of CVD for every additional 4 eggs/week (40% in those with unspecified diabetes).
Rong, 2013 [60]	SRMA of PCS	8 (263,938)	CHD, stroke	No linear association for CHD risk. No DR effect. In those with unspecified diabetes, high egg consumers had 54% increased risk of CHD. No linear association or DR effect for stroke.
Shin, 2013 [52]	SRMA of PCS	8 (348,420)	CVD, IHD, stroke	No associations for CVD, IHD or stroke incidence when comparing intakes of <1/week with ≥1/day. In those with unspecified diabetes, high egg consumers had 69% increased risk of CVD.
Alexander, 2016 [61]	SRMA of PCS	10 (not stated)	CHD, stroke	No associations for CHD incidence when comparing intakes of <2/week with ≥1/day. 12% reduced risk of stroke in high egg consumers when comparing intakes of <2/week with ≥1/day.
Xu, 2018 [55]	SRMA of PCS	9 (not stated)	Stroke, IHD, CVD	No significant associations for IHD risk, CVD mortality, IHD mortality or stroke mortality and egg consumption. 9% reduced risk of stroke in high egg consumers when comparing <1/week with ≥7/week.
Drouin-Chartier, 2020 [53]	SRMA of PCS	28 (1,720,108)	CVD, stroke, CHD	No significant associations for CVD, stroke or CHD at egg intakes of 1/day compared with lower intakes. In those with T2D, high egg consumers had 40% increased risk of CVD. DR analysis in T2D: 25% increased risk for each additional egg consumed per day.
Krittanawong, 2020 [54]	SRMA of PCS	23 (1,415,839)	CVD, stroke, CAD	No significant associations for risk of CVD. 11% reduced risk of CAD in higher egg consumers when comparing >1/day) to ≤1/day). No associations with risk of stroke.
Tang, 2020 [62]	SRMA of PCS	16 (not stated)	Stroke	No significant association between egg intake and risk of stroke but borderline reduced risk in high consumers for stroke mortality. DR analysis: non-linear association between egg consumption and risk of stroke; 1–4/week associated with decreased risk while >10/week associated with increased risk.
Djoussé, 2021 [63]	Pooled analysis and MA of PCS	7 (not stated)	CHD	No significant association for risk of CHD even in those with T2D at intakes up to 7+ eggs/week.
Godos, 2021 [49]	SRMA of PCS	39 (1,831,083)	CVD, CHD, stroke, heart failure	4% decreased risk of CVD events/mortality in high egg consumers eating 1–6/week vs. non-consumers. <2 eggs/week associated with 4% decreased risk of CHD incidence and mortality. No association for risk of stroke events or mortality. For heart failure, 15% increased risk at ≤7 eggs/week and 23% increased risk at 9 eggs/week vs. non-consumers.
Zhao, 2022 [51]	SRMA of PCS	41 (3,601,401)	CVD	4% increased risk of CVD for each additional egg/day consumed vs. non-consumers.
Darooghegi Mofrad, 2022 [56]	SRMA of PCS	16 (1,479,181)	CVD	No significant associations for CVD mortality at egg intakes of 1/day vs. 0.007/day and no DR effect.
Ma, 2022 [57]	SRMA of PCS	14 (not stated)	CVD, IHD, stroke	No significant associations with risk of mortality for CVD, IHD or stroke when comparing high vs. low egg consumption. No DR effects per additional 1 egg/day.
Yang, 2022 [59]	SRMA of PCS	9 (943,827)	CVD, stroke	7% increased risk of CVD mortality with each additional egg/day. No significant associations for stroke mortality.
Mousavi, 2022 [58]	SRMA of PCS	32 (2,216,720)	CVD, CHD, stroke	No significant associations with risk of mortality for CVD, CHD or stroke. DR analysis: for each additional 1 egg/week, risk of stroke mortality decreased by 4%.

Key: CAD, coronary artery disease; CHD, coronary heart disease; CVD, cardiovascular disease; DR, dose-response; IHD, ischaemic heart disease; SRMA, systematic review and meta-analysis; MA, meta-analysis; PCS, prospective cohort studies; T2D, type 2 diabetes mellitus.

**Table 5:** Observational studies on eggs and metabolic risk markers.

Author, Year	Study Design	Number of Studies (Number of Participants)	Outcomes	Significant Differences in Relative Risk between Highest and Lowest Egg Consumers
Li, 2013 [50]	SRMA of PCS	7 (64,447)	Unspecified DM	High consumers had 68% increased risk of developing T2D. DR analysis: for each additional 4 eggs/week, risk of DM 29% greater.
Shin, 2013 [52]	SRMA of PCS	3 (69,297)	T2D	High consumers had 42% increased risk of T2D (≥1 egg/day vs. <1 egg/week).
Djoussé, 2016 [65]	SRMA of PCS	12 (219,979)	T2D	High consumers had 9% increased risk of developing T2D. DR analysis: elevated risk of 7% only when >5 eggs/week consumed.
Tamez, 2016 [66]	SRMA of PCS	10 (251,213)	T2D	DR analysis: each additional egg per day associated with 13% higher risk of T2D.
Drouin-Chartier, 2020 [67]	SRMA of PCS	16 (589,559)	T2D	DR analysis: each additional egg per day associated with 7% higher risk of T2D.
Djoussé, 2021 [63]	Pooled analysis and MA of PCS	9 (103,811)	T2D	No association with T2D risk at 1 egg/week ≥2 eggs/week associated with increased risk (11–27% depending on intake) vs. zero intake.

Key: DM, diabetes mellitus; DR, dose-response; MA, meta-analysis; SRMA, systematic review and meta-analysis; T2D, type 2 diabetes mellitus.

**Table 7:** Intervention studies on eggs and markers of weight management.

Author, Year	Study Design	Intervention	Number of Participants	Outcomes	Results
Ratliff, 2010 [77]	RCT, crossover	Egg breakfast vs. bagel breakfast	21 men (20–70 y)	Satiety, appetite hormones, EI	EI at lunch (−112 kcal) and over 24 h (−403 kcal) significantly lower after eggs Vs. bagel. Serum ghrelin and hunger scores significantly lower and satiety scores higher after eggs vs. bagel.
Pombo-Rodrigues, 2011 [78]	RCT, crossover	Omelette vs. jacket potato vs. chicken lunch	31 adults (37.5 ± 9.97 y)	Satiety, EI	After eggs, significantly lower desire to eat, greater fullness score and lower desire to eat vs. jacket potato. No significant differences in later EI.
Fallaize, 2012 [79]	RCT, crossover	Egg breakfast vs. cereal breakfast vs. croissant breakfast	30 men (21.7 ± 1.2 y)	Satiety, EI	After eggs, increased satiety, less hunger and lower desire to eat vs. cereal or croissant. EI at lunch lower (−158 kcal) after eggs vs. croissant. EI at evening meal lower (−315 kcal) after eggs vs. cereal.
Liu, 2015 [80]	RCT, crossover	Egg breakfast vs. bagel breakfast	13 children (5 y) 15 adolescents (15.6 ± 1.1 y)	Satiety, appetite hormones, EI	No differences between test breakfasts for EI or reported appetite ratings for any age group. PYY increased significantly 180 min after eggs compared with bagel in adolescents.
Fuller, 2015 [81]	SBRCT, parallel	High egg diet (12/week) vs. low egg diet (<2/week) (breakfast matched for protein) for 3 months	140 adults with T2D (49–69 y)	Satiety	Those on high egg diet reported significantly less hunger and greater satiety post-breakfast compared with low egg diet.
Bonnema, 2016 [82]	RCT, crossover	3 test breakfasts: low egg/high fibre vs. high egg/low fibre vs. cereal low protein/low fibre	48 adults (24 ± 1 y)	Satiety, EI	Reported satiety and satisfaction higher and reported hunger and prospective food intake score lower after high egg breakfasts vs. others. EI at lunch lower after both egg breakfasts compared with cereal breakfast.
Kral, 2016 [83]	RCT, crossover	Egg vs. cereal vs. oatmeal breakfast	40 children (8–10 y)	EI, satiety	EI at lunch lower (−70 kcal) after egg breakfast vs. other breakfasts. EI not significant differences between breakfasts for energy consumed over the remainder of the test day or in appetite ratings over time.
Fuller, 2018 [76]	RCT, parallel	High egg breakfast (12/week) vs. low egg diet (<2/week) + energy restriction, for 3 months	128 adults at risk of T2D or with confirmed T2D (49–71 y)	Weight	No significant differences in weight loss between high egg vs. low egg diets.
Zhu, 2022 [84]	RCT, crossover	Egg breakfast vs. cereal breakfast (matched for protein) + energy restriction for 7 d	60 females (24 ± 4.9 y)	Satiety, appetite hormones, EI	No significant differences in EI or appetite hormones between diet groups. Reported fullness significantly greater after eggs.

Key: d, day; EI, energy intake; h, hours; PYY, peptide YY; RCT, randomised controlled trial; T2D, type 2 diabetes mellitus, vs., versus; y, years.

## Data Availability

Data sharing not applicable. No new data were created or analysed in this study. Data sharing is not applicable to this article.

## References

[1] Gray J., Griffin B. (2009). Eggs and dietary cholesterol—Dispelling the myth. Nutr. Bull..

[2] Hooper L., Martin N., Jimoh O.F., Kirk C., Foster E., Abdelhamid A.S. (2020). Reduction in saturated fat intake for cardiovascular disease. Cochrane Database Syst. Rev..

[3] U.S. Department of Health and Human Services 2015–2020 Dietary Guidelines. https://health.gov/our-work/nutrition-physical-activity/dietary-guidelines/previous-dietary-guidelines/2015.

[4] Patrick M.E., Adcock P.M., Gomez T.M., Altekruse S.F., Holland B.H., Tauxe R.V., Swerdlow D.L. (2004). *Salmonella* Enteritidis Infections, United States, 1985–1999. Emerg. Infect. Dis..

[5] Advisory Committee on the Microbiological Safety of Food Ad Hoc Group on Eggs An Update on the Microbiological Risk from Shell Eggs and Their Products. https://acmsf.food.gov.uk/sites/default/files/acmsf-egg-reportv1.pdf.

[6] European Commission Food-Based Dietary Guidelines in Europe—Table 10. https://knowledge4policy.ec.europa.eu/health-promotion-knowledge-gateway/food-based-dietary-guidelines-europe-table-10_en.

[7] Walker S., Baum J.I. (2022). Eggs as an affordable source of nutrients for adults and children living in food-insecure environments. Nutr. Rev..

[8] OECD iLibrary Table C.47—Egg projections: Production and Food Consumption. https://www.oecd-ilibrary.org/agriculture-and-food/egg-projections-production-and-food-consumption_0227d3e6-en.

[9] International Egg Commission Global Egg Production Continues to Increase at an Average of 3% per Year. https://www.internationalegg.com/resource/global-egg-production-continues-to-increase-at-an-average-of-3-per-year/.

[10] Industry Data on Population Egg Consumption. https://www.egginfo.co.uk/egg-facts-and-figures/industry-information/data.

[11] Public Health England McCance and Widdowson’s Composition of Foods Integrated Dataset. https://www.gov.uk/government/publications/composition-of-foods-integrated-dataset-cofid.

[12] Griffin B. (2016). Eggs: Good or bad?. Proc. Nutr. Soc..

[13] McNamara D.J., Caballero B. (2013). Eggs. Encyclopedia of Human Nutrition.

[14] European Commission Regulation (EU) No 1169/2011 of the European Parliament and of the Council of 25 October 2011 on the Provision of Food Information to Consumers. https://eur-lex.europa.eu/LexUriServ/LexUriServ.do?uri=OJ:L:2011:304:0018:0063:en:PDF.

[15] European Parliament and Council (2007). Regulation (EC) No 1924/2006 on Nutrition and Health Claims made on Foods. OJEU.

[16] European Commission EU Register of Health Claims. https://ec.europa.eu/food/food-feed-portal/screen/health-claims/eu-register.

[17] European Food Safety Authority (2011). Scientific opinion on the substantiation of health claims related to choline and contribution to normal lipid metabolism (ID 3186), maintenance of normal liver function (ID 1501), contribution to normal homocysteine metabolism (ID 3090), maintenance of normal neurological function (ID 1502), contribution to normal cognitive function (ID 1502), and brain and neurological development (ID 1503) pursuant to Article 13(1) of Regulation (EC) No 1924/2006. EFSA J..

[18] Turck D., Bohn T., Castenmiller J., de Henauw S., Hirsch-Ernst K.I., Knutsen H.K., Maciuk A., Mangelsdorf I., McArdle H.J., EFSA NDA Panel (EFSA Panel on Nutrition, Novel Foods and Food Allergens) (2022). Scientific Opinion on the scientific advice related to nutrient profiling for the development of harmonised mandatory front-of-pack nutrition labelling and the setting of nutrient profiles for restricting nutrition and health claims on foods. EFSA J..

[19] Wiedeman A.M., Barr S.I., Green T.J., Xu Z., Innis S.M., Kitts D.D. (2018). Dietary choline intake: Current state of knowledge across the life cycle. Nutrients.

[20] EFSA Panel on Dietetic Products, Nutrition and Allergies (NDA) (2016). Dietary Reference Values for choline. EFSA J..

[21] Institute of Medicine (US) Standing Committee on the Scientific Evaluation of Dietary Reference Intakes and its Panel on Folate, Other B Vitamins, and Choline (1998). Dietary Reference Intakes: Thiamin, Riboflavin, Niacin, Vitamin B6, Folate, Vitamin B12, Pantothenic Acid, Biotin, and Choline.

[22] Vennemann F.B.C., Ioannidou S., Valsta L.M., Dumas C., Ocké M.C., Mensink G.B.M., Lindtner O., Virtanen S.M., Tlustos C., D’addezio L. (2015). Dietary intake and food sources of choline in European populations. Br. J. Nutr..

[23] Wallace T.C., Fulgoni V.L. (2017). Usual Choline Intakes Are Associated with Egg and Protein Food Consumption in the United States. Nutrients.

[24] Caudill M.A., Strupp B.J., Muscalu L., Nevins J.E.H., Canfield R.L. (2018). Maternal choline supplementation during the third trimester of pregnancy improves infant information processing speed: A randomized, double-blind, controlled feeding study. FASEB J..

[25] Bahnfleth C.L., Strupp B.J., Caudill M.A., Canfield R.L. (2022). Prenatal choline supplementation improves child sustained attention: A 7-year follow-up of a randomized controlled feeding trial. FASEB J..

[26] Probst Y., Sulistyoningrum D.C., Netting M.J., Gould J.F., Wood S., Makrides M., Best K.P., Green T.J. (2022). Estimated Choline Intakes and Dietary Sources of Choline in Pregnant Australian Women. Nutrients.

[27] Zhu C., Sawrey-Kubicek L., Bardagjy A.S., Houts H., Tang X., Sacchi R., Randolph J.M., Steinberg F.M., Zivkovic A.M. (2020). Whole egg consumption increases plasma choline and betaine without affecting TMAO levels or gut microbiome in overweight postmenopausal women. Nutr. Res..

[28] Huotari A., Herzig K.H. (2008). Vitamin D and living in northern latitudes--an endemic risk area for vitamin D deficiency. Int. J. Circumpolar Health.

[29] Rippin H.L., Hutchinson J., Jewell J., Breda J.J., Cade J.E. (2017). Adult Nutrient Intakes from Current National Dietary Surveys of European Populations. Nutrients.

[30] Public Health England Government Dietary Recommendations. https://assets.publishing.service.gov.uk/government/uploads/system/uploads/attachment_data/file/618167/government_dietary_recommendations.pdf.

[31] Food Safety Authority of Ireland Vitamin D: Scientific Recommendations for 5 to 65 Year Olds Living in Ireland. https://www.fsai.ie/news_centre/press_releases/vitamin_D_report_14022023.html.

[32] Daly R.M., De Ross B., Gianoudis J., Tan S.Y. (2022). Dose-Response Effect of Consuming Commercially Available Eggs on Wintertime Serum 25-Hydroxyvitamin D Concentrations in Young Australian Adults: A 12-Week Randomized Controlled Trial. J. Nutr..

[33] Public Health England National Diet and Nutrition Survey Rolling Programme Years 9 to 11 (2016/2017 to 2018/2019). https://assets.publishing.service.gov.uk/government/uploads/system/uploads/attachment_data/file/943114/NDNS_UK_Y9-11_report.pdf.

[34] Dorrington N., Fallaize R., Hobbs D.A., Weech M., Lovegrove J.A. (2020). A Review of Nutritional Requirements of Adults Aged ≥65 Years in the UK. J. Nutr..

[35] Rotstein A., Kodesh A., Goldberg Y., Reichenberg A., Levine S.Z. (2022). Serum folate deficiency and the risks of dementia and all-cause mortality: A national study of old age. Evid. Based Ment. Health.

[36] Ruxton C.H.S., Derbyshire E., Gibson S. (2010). The nutritional properties and health benefits of eggs. Nutr. Food Sci..

[37] Gibson S., Gray J. (2020). Evaluating current egg consumption patterns: Associations with diet quality, nutrition and health status in the UK National Diet and Nutrition Survey. Nutr. Bull..

[38] World Health Organisation (2007). Assessment of Iodine Deficiency Disorders and Monitoring Their Elimination, A Guide for Programme Managers.

[39] Abel M.H., Caspersen I.H., Sengpiel V., Jacobsson B., Meltzer H.M., Magnus P., Alexander J., Brantsæter A.L. (2020). Insufficient maternal iodine intake is associated with subfecundity, reduced foetal growth, and adverse pregnancy outcomes in the Norwegian Mother, Father and Child Cohort Study. BMC Med..

[40] Abel M.H., Caspersen I.H., Meltzer H.M., Haugen M., Brandlistuen R.E., Aase H., Alexander J., Torheim L.E., Brantsæter A.L. (2017). Suboptimal Maternal Iodine Intake Is Associated with Impaired Child Neurodevelopment at 3 Years of Age in the Norwegian Mother and Child Cohort Study. J. Nutr..

[41] Tome D. (2012). Criteria and markers for protein quality assessment—A review. Br. J. Nutr..

[42] Donaldson A.I.C., Johnstone A.M., de Roos B., Myint P.K. (2018). Role of protein in healthy ageing. Eur. J. Integr. Med..

[43] EFSA Panel on Dietetic Products, Nutrition and Allergies (NDA) (2012). Scientific Opinion on Dietary Reference Values for protein. EFSA J.

[44] Rouhani M.H., Rashidi-Pourfard N., Salehi-Abargouei A., Karimi M., Haghighatdoost F. (2018). Effects of Egg Consumption on Blood Lipids: A Systematic Review and Meta-Analysis of Randomized Clinical Trials. J. Am. Coll. Nutr..

[45] Wang M.X., Wong C.H., Kim J.E. (2019). Impact of whole egg intake on blood pressure, lipids and lipoproteins in middle-aged and older population: A systematic review and meta-analysis of randomized controlled trials. Nutr. Metab. Cardiovasc. Dis..

[46] Li M.Y., Chen J.H., Chen C., Kang Y.N. (2020). Association between Egg Consumption and Cholesterol Concentration: A Systematic Review and Meta-analysis of Randomized Controlled Trials. Nutrients.

[47] Khalighi Sikaroudi M., Soltani S., Kolahdouz-Mohammadi R., Clayton Z.S., Fernandez M.L., Varse F., Shidfar F. (2020). The responses of different dosages of egg consumption on blood lipid profile: An updated systematic review and meta-analysis of randomized clinical trials. J. Food Biochem..

[48] Calling S., Johansson S.E., Wolff M., Sundquist J., Sundquist K. (2021). Total cholesterol/HDL-C ratio versus non-HDL-C as predictors for ischemic heart disease: A 17-year follow-up study of women in southern Sweden. BMC Cardiovasc. Disord..

[49] Godos J., Micek A., Brzostek T., Toledo E., Iacoviello L., Astrup A., Franco O.H., Galvano F., Martinez-Gonzalez M.A., Grosso G. (2021). Egg consumption and cardiovascular risk: A dose-response meta-analysis of prospective cohort studies. Eur. J. Nutr..

[50] Li Y., Zhou C., Zhou X., Li L. (2013). Egg consumption and risk of cardiovascular diseases and diabetes: A meta-analysis. Atherosclerosis.

[51] Zhao B., Gan L., Graubard B.I., Männistö S., Albanes D., Huang J. (2022). Associations of Dietary Cholesterol, Serum Cholesterol, and Egg Consumption With Overall and Cause-Specific Mortality: Systematic Review and Updated Meta-Analysis. Circulation.

[52] Shin J.Y., Xun P., Nakamura Y., He K. (2013). Egg consumption in relation to risk of cardiovascular disease and diabetes: A systematic review and meta-analysis. Am. J. Clin. Nutr..

[53] Drouin-Chartier J.P., Chen S., Li Y., Schwab A.L., Stampfer M.J., Sacks F.M., Rosner B., Willett W.C., Hu F.B., Bhupathiraju S.N. (2020). Egg consumption and risk of cardiovascular disease: Three large prospective US cohort studies, systematic review, and updated meta-analysis. BMJ.

[54] Krittanawong C., Narasimhan B., Wang Z., Virk H.U.H., Farrell A.M., Zhang H., Tang W.H.W. (2021). Association Between Egg Consumption and Risk of Cardiovascular Outcomes: A Systematic Review and Meta-Analysis. Am. J. Med..

[55] Xu L., Lam T.H., Jiang C.Q., Zhang W.S., Zhu F., Jin Y.L., Woo J., Cheng K.K., Thomas G.N. (2019). Egg consumption and the risk of cardiovascular disease and all-cause mortality: Guangzhou Biobank Cohort Study and meta-analyses. Eur. J. Nutr..

[56] Darooghegi Mofrad M., Naghshi S., Lotfi K., Beyene J., Hypponen E., Pirouzi A., Sadeghi O. (2022). Egg and Dietary Cholesterol Intake and Risk of All-Cause, Cardiovascular, and Cancer Mortality: A Systematic Review and Dose-Response Meta-Analysis of Prospective Cohort Studies. Front. Nutr..

[57] Ma W., Zhang Y., Pan L., Wang S., Xie K., Deng S., Wang R., Guo C., Qin P., Wu X. (2022). Association of Egg Consumption with Risk of All-Cause and Cardiovascular Disease Mortality: A Systematic Review and Dose–Response Meta-Analysis of Observational Studies. J. Nutr..

[58] Mousavi S.M., Zargarzadeh N., Rigi S., Persad E., Pizarro A.B., Hasani-Ranjbar S., Larijani B., Willett W.C., Esmaillzadeh A. (2022). Egg Consumption and Risk of All-Cause and Cause-Specific Mortality: A Systematic Review and Dose-Response Meta-analysis of Prospective Studies. Adv. Nutr..

[59] Yang P.F., Wang C.R., Hao F.B., Peng Y., Wu J.J., Sun W.P., Hu J.J., Zhong G.C. (2022). Egg consumption and risks of all-cause and cause-specific mortality: A dose-response meta-analysis of prospective cohort studies. Nutr. Rev..

[60] Rong Y., Chen L., Zhu T., Song Y., Yu M., Shan Z., Sands A., Hu F.B., Liu L. (2013). Egg consumption and risk of coronary heart disease and stroke: Dose-response meta-analysis of prospective cohort studies. BMJ.

[61] Alexander D.D., Miller P.E., Vargas A.J., Weed D.L., Cohen S.S. (2016). Meta-analysis of Egg Consumption and Risk of Coronary Heart Disease and Stroke. J. Am. Coll. Nutr..

[62] Tang H., Cao Y., Yang X., Zhang Y. (2020). Egg Consumption and Stroke Risk: A Systematic Review and Dose-Response Meta-Analysis of Prospective Studies. Front. Nutr..

[63] Djoussé L., Zhou G., McClelland R., Ma N., Zhou X., Kabagambe E.K., Talegawkar S., Judd S.E., Biggs M.L., Fitzpatrick A. (2021). Egg consumption, overall diet quality, and risk of type 2 diabetes and coronary heart disease: A pooling project of US prospective cohorts. Clin. Nutr..

[64] Diabetes UK. Differences between Type 1 and Type 2 Diabetes. https://www.diabetes.org.uk/diabetes-the-basics/differences-between-type-1-and-type-2-diabetes.

[65] Djoussé L., Khawaja O.A., Gaziano J.M. (2016). Egg consumption and risk of type 2 diabetes: A meta-analysis of prospective studies. Am. J. Clin. Nutr..

[66] Tamez M., Virtanen J.K., Lajous M. (2016). Egg consumption and risk of incident type 2 diabetes: A dose-response meta-analysis of prospective cohort studies. Am. J. Clin. Nutr..

[67] Drouin-Chartier J.-P., Schwab A.L., Chen S., Li Y., Sacks F.M., Rosner B., Manson J.E., Willett W.C., Stampfer M.J., Hu F.B. (2020). Egg consumption and risk of type 2 diabetes: Findings from 3 large US cohort studies of men and women and a systematic review and meta-analysis of prospective cohort studies. Am. J. Clin. Nutr..

[68] Ding J., Zhang Y. (2022). Relationship between Egg Consumption and Metabolic Syndrome. A Meta-Analysis of Observational Studies. J. Nutr. Health Aging.

[69] Andersen C.J., Blesso C.N., Lee J., Barona J., Shah D., Thomas M.J., Fernandez M.L. (2013). Egg consumption modulates HDL lipid composition and increases the cholesterol-accepting capacity of serum in metabolic syndrome. Lipids.

[70] Blesso C.N., Andersen C.J., Bolling B.W., Fernandez M.L. (2013). Egg intake improves carotenoid status by increasing plasma HDL cholesterol in adults with metabolic syndrome. Food Funct..

[71] Blesso C.N., Andersen C.J., Barona J., Volek J.S., Fernandez M.L. (2013). Whole egg consumption improves lipoprotein profiles and insulin sensitivity to a greater extent than yolk-free egg substitute in individuals with metabolic syndrome. Metabolism.

[72] Thomas M.S., Puglisi M., Malysheva O., Caudill M.A., Sholola M., Cooperstone J.L., Fernandez M.L. (2022). Eggs Improve Plasma Biomarkers in Patients with Metabolic Syndrome Following a Plant-Based Diet-A Randomized Crossover Study. Nutrients.

[73] Thomas M.S., Huang L., Garcia C., Sakaki J.R., Blesso C.N., Chun O.K., Fernandez M.L. (2022). The Effects of Eggs in a Plant-Based Diet on Oxidative Stress and Inflammation in Metabolic Syndrome. Nutrients.

[74] Thomas M.S., DiBella M., Blesso C.N., Malysheva O., Caudill M., Sholola M., Cooperstone J.L., Fernandez M.L. (2022). Comparison between Egg Intake versus Choline Supplementation on Gut Microbiota and Plasma Carotenoids in Subjects with Metabolic Syndrome. Nutrients.

[75] Wilcox J., Skye S.M., Graham B., Zabell A., Li X.S., Li L., Shelkay S., Fu X., Neale S., O’Laughlin C. (2021). Dietary Choline Supplements, but Not Eggs, Raise Fasting TMAO Levels in Participants with Normal Renal Function: A Randomized Clinical Trial. Am. J. Med..

[76] Fuller N.R., Sainsbury A., Caterson I.D., Denyer G., Fong M., Gerofi J., Leung C., Lau N.S., Williams K.H., Januszewski A.S. (2018). Effect of a high-egg diet on cardiometabolic risk factors in people with type 2 diabetes: The Diabetes and Egg (DIABEGG) Study—randomized weight-loss and follow-up phase. Am. J. Clin. Nutr..

[77] Ratliff J., Leite J.O., de Ogburn R., Puglisi M.J., VanHeest J., Fernandez M.L. (2010). Consuming eggs for breakfast influences plasma glucose and ghrelin, while reducing energy intake during the next 24 hours in adult men. Nutr. Res..

[78] Pombo-Rodrigues S., Calame W., Re R. (2011). The effects of consuming eggs for lunch on satiety and subsequent food intake. Int. J. Food Sci. Nutr..

[79] Fallaize R., Wilson L., Gray J., Morgan L.M., Griffin B.A. (2012). Variation in the effects of three different breakfast meals on subjective satiety and subsequent intake of energy at lunch and evening meal. Eur. J. Nutr..

[80] Liu A.G., Puyau R.S., Han H., Johnson W.D., Greenway F.L., Dhurandhar N.V. (2015). The effect of an egg breakfast on satiety in children and adolescents: A randomized crossover trial. J. Am. Coll. Nutr..

[81] Fuller N.R., Caterson I.D., Sainsbury A., Denyer G., Fong M., Gerofi J., Baqleh K., Williams K.H., Lau N.S., Markovic T.P. (2015). The effect of a high-egg diet on cardiovascular risk factors in people with type 2 diabetes: The Diabetes and Egg (DIABEGG) study-a 3-mo randomized controlled trial. Am. J. Clin. Nutr..

[82] Bonnema A.L., Altschwager D.K., Thomas W., Slavin J.L. (2016). The effects of the combination of egg and fiber on appetite, glycemic response and food intake in normal weight adults—A randomized, controlled, crossover trial. Int. J. Food Sci. Nutr..

[83] Kral T.V., Bannon A.L., Chittams J., Moore R.H. (2016). Comparison of the satiating properties of egg- versus cereal grain-based breakfasts for appetite and energy intake control in children. Eat. Behav..

[84] Zhu Y., Bailey D., Childress A., Dawson J.A., Binks M., Dhurandhar N.V. (2022). Greater protein quality of an egg breakfast may be inadequate to induce satiety during weight loss, compared with a cereal breakfast of equal protein quantity. Int. J. Food Sci. Nutr..

[85] van Vliet S., Shy E.L., Sawan S.A., Beals J.W., West D.W., Skinner S.K., Ulanov A.V., Li Z., Paluska S.A., Parsons C.M. (2017). Consumption of whole eggs promotes greater stimulation of postexercise muscle protein synthesis than consumption of isonitrogenous amounts of egg whites in young men. Am. J. Clin. Nutr..

[86] Bagheri R., Moghadam B.H., Ashtary-Larky D., Forbes S.C., Candow D.G., Galpin A.J., Eskandari M., Kreider R.B., Wong A. (2021). Whole Egg Vs. Egg White Ingestion During 12 weeks of Resistance Training in Trained Young Males: A Randomized Controlled Trial. J. Strength Cond. Res..

[87] Bagheri R., Moghadam B.H., Jo E., Tinsley G.M., Stratton M.T., Ashtary-Larky D., Eskandari M., Wong A. (2020). Comparison of whole egg v. egg white ingestion during 12 weeks of resistance training on skeletal muscle regulatory markers in resistance-trained men. Br. J. Nutr..

[88] Witard O.C., Bannock L., Tipton K.D. (2022). Making Sense of Muscle Protein Synthesis: A Focus on Muscle Growth During Resistance Training. Int. J. Sport Nutr. Exerc. Metab..

[89] Hida A., Hasegawa Y., Mekata Y., Usuda M., Masuda Y., Kawano H., Kawano Y. (2012). Effects of egg white protein supplementation on muscle strength and serum free amino acid concentrations. Nutrients.

[90] Park S., Church D.D., Schutzler S.E., Azhar G., Kim I.Y., Ferrando A.A., Wolfe R.R. (2021). Metabolic Evaluation of the Dietary Guidelines’ Ounce Equivalents of Protein Food Sources in Young Adults: A Randomized Controlled Trial. J. Nutr..

[91] Kim I.Y., Shin Y.A., Schutzler S.E., Azhar G., Wolfe R.R., Ferrando A.A. (2018). Quality of meal protein determines anabolic response in older adults. Clin. Nutr..

[92] Ullevig S.L., Zuniga K., Austin Lobitz C., Santoyo A., Yin Z. (2022). Egg protein supplementation improved upper body muscle strength and protein intake in community-dwelling older adult females who attended congregate meal sites or adult learning centers: A pilot randomized controlled trial. Nutr. Health.

[93] Wright C.S., Zhou J., Sayer R.D., Kim J.E., Campbell W.W. (2018). Effects of a High-Protein Diet Including Whole Eggs on Muscle Composition and Indices of Cardiometabolic Health and Systemic Inflammation in Older Adults with Overweight or Obesity: A Randomized Controlled Trial. Nutrients.

[94] World Wildlife Fund (WWF) Eating for 2 Degrees New and Updated Livewell Plates Summary Report. https://www.wwf.org.uk/sites/default/files/2017-09/WWF_Livewell_Plates_Summary_Report_Sept2017_Web.pdf.

[95] Poore J., Nemecek T. (2018). Reducing food’s environmental impacts through producers and consumers. Science.

[96] The National Food Strategy: The Plan—July 2021. https://assets.publishing.service.gov.uk/government/uploads/system/uploads/attachment_data/file/1025825/national-food-strategy-the-plan.pdf.

[97] British Dietetic Association One Blue Dot—The BDA’s Environmentally Sustainable Diet Project. https://www.bda.uk.com/resource/one-blue-dot.html.

[98] EAT Forum The EAT-Lancet Commission on Food, Planet, Health. https://eatforum.org/eat-lancet-commission/.

[99] Our World in Data Greenhouse Gas Emissions Per 100 Grams of Protein. https://ourworldindata.org/grapher/ghg-per-protein-poore.

[100] World Resources Institute (WRI) Creating a Sustainable Food Future. https://research.wri.org/sites/default/files/2019-07/WRR_Food_Full_Report_0.pdf.

[101] Clune S., Crossin E., Verghese K. (2016). Systematic review of greenhouse gas emissions for different fresh food categories. J. Clean. Prod..

[102] van Vliet S., Kronberg S.L., Provenza F.D. (2020). Plant-Based Meats, Human Health, and Climate Change. Front. Sustain. Food Syst..

[103] Gehring J., Touvier M., Baudry J., Julia C., Buscail C., Srour B., Hercberg S., Péneau S., Kesse-Guyot E., Allès B. (2021). Consumption of Ultra-Processed Foods by Pesco-Vegetarians, Vegetarians, and Vegans: Associations with Duration and Age at Diet Initiation. J. Nutr..

[104] YouGov What Is Making Flexitarians in the US and UK Shift Towards a Meatless Diet?. https://yougov.co.uk/topics/consumer/articles-reports/2021/05/31/what-making-flexitarians-us-and-uk-shift-towards-m.

[105] Longo G., Berti I., Burks A.W., Krauss B., Barbi E. (2013). IgE-mediated food allergy in children. Lancet.

[106] Du Toit G., Roberts G., Sayre P.H., Bahnson H.T., Radulovic S., Santos A.F., Brough H.A., Phippard D., Basting M., Feeney M. (2015). Randomized Trial of Peanut Consumption in Infants at Risk for Peanut Allergy. N. Engl. J. Med..

[107] Perkin M.R., Logan K., Marrs T., Radulovic S., Craven J., Flohr C., Lack G., Young L., Offord V., DeSousa M. (2016). Enquiring About Tolerance (EAT) study: Feasibility of an early allergenic food introduction regimen. J. Allergy Clin. Immunol..

[108] Natsume O., Kabashima S., Nakazato J., Yamamoto-Hanada K., Narita M., Kondo M., Saito M., Kishino A., Takimoto T., Inoue E. (2017). Two-step egg introduction for prevention of egg allergy in high-risk infants with eczema (PETIT): A randomised, double-blind, placebo-controlled trial. Lancet.

[109] Wen X., Martone G.M., Lehman H.K., Rideout T.C., Cameron C.E., Dashley S., Konnayil B.J. (2023). Frequency of Infant Egg Consumption and Risk of Maternal-Reported Egg Allergy at 6 Years. J. Nutr..

[110] Public Health England and Committee on Toxicity of Chemicals in Food, Consumer Products and the Environment SACN-COT Statement on the Introduction of Peanut and Hen’s Egg into the Infant Diet. https://www.gov.uk/government/publications/sacn-cot-statement-on-the-introduction-of-peanut-and-hens-egg-into-the-infant-diet.

[111] Ierodiakonou D., Larsen V.G., Logan A., Groome A., Cunha S., Chivinge J., Robinson Z., Geoghegan N., Jarrold K., Reeves T. (2016). Timing of Allergenic Food Introduction to the Infant Diet and Risk of Allergic or Autoimmune Disease: A Systematic Review and Meta-analysis. JAMA.

[112] BSACI/British Dietetic Association Food Allergy Specialist Group Infant Feeding and Allergy Prevention Guidance for Parents. https://www.bsaci.org/wp-content/uploads/2020/02/pdf_Infant-feeding-and-allergy-prevention-PARENTS-FINAL-booklet.pdf.

[113] Department for Environment, Food & Rural Affairs and Public Health England Zoonoses Report UK 2012. https://assets.publishing.service.gov.uk/government/uploads/system/uploads/attachment_data/file/236983/pb13987-zoonoses-report-2012.pdf.

[114] Egg Info British Lion Eggs. https://www.egginfo.co.uk/british-lion-eggs.

[115] The National Archives, Food Standards Agency New Advice on Eating Runny Eggs. https://webarchive.nationalarchives.gov.uk/ukgwa/20171207160203/https:/www.food.gov.uk/news-updates/news/2017/16597/new-advice-on-eating-runny-eggs.

[116] Young S.S., Karr A. (2011). Deming, data and observational studies. A process out of control and needing fixing. Significance.

[117] Ruxton C. (2022). Interpretation of observational studies: The good, the bad and the sensational. Proc. Nutr. Soc..

[118] Barraj L., Tran N., Mink P. (2009). A comparison of egg consumption with other modifiable coronary heart disease lifestyle risk factors: A relative risk apportionment study. Risk Anal..

[119] Missimer A., Fernandez M.L., DiMarco D.M., Norris G.H., Blesso C.N., Murillo A.G., Vergara-Jimenez M., Lemos B.S., Medina-Vera I., Malysheva O.V. (2017). Compared to an Oatmeal Breakfast, Two Eggs/Day Increased Plasma Carotenoids and Choline without Increasing Trimethyl Amine *N*-Oxide Concentrations. J. Am. Coll. Nutr..

[120] Miller C.A., Corbin K.D., da Costa K.A., Zhang S., Zhao X., Galanko J.A., Blevins T., Bennett B.J., O’Connor A., Zeisel S.H. (2014). Effect of egg ingestion on trimethylamine-N-oxide production in humans: A randomized, controlled, dose-response study. Am. J. Clin. Nutr..

[121] McDonald J.D., Mah E., Chitchumroonchokchai C., Reverri E.J., Li J., Volek J.S., Villamena F.A., Bruno R.S. (2018). Co-ingestion of whole eggs or egg whites with glucose protects against postprandial hyperglycaemia-induced oxidative stress and dysregulated arginine metabolism in association with improved vascular endothelial function in prediabetic men. Br. J. Nutr..

[122] Gylling H., Hallikainen M., Pihlajamäki J., Simonen P., Kuusisto J., Laakso M., Miettinen T.A. (2010). Insulin sensitivity regulates cholesterol metabolism to a greater extent than obesity: Lessons from the METSIM Study. J. Lipid Res..

[123] Knopp R.H., Retzlaff B., Fish B., Walden C., Wallick S., Anderson M., Aikawa K., Kahn S.E. (2003). Effects of insulin resistance and obesity on lipoproteins and sensitivity to egg feeding. Arter. Thromb. Vasc. Biol..

[124] Pearce K.L., Clifton P.M., Noakes M. (2011). Egg consumption as part of an energy-restricted high-protein diet improves blood lipid and blood glucose profiles in individuals with type 2 diabetes. Br. J. Nutr..

[125] Richard C., Cristall L., Fleming E., Lewis E.D., Ricupero M., Jacobs R.L., Field C.J. (2017). Impact of Egg Consumption on Cardiovascular Risk Factors in Individuals with Type 2 Diabetes and at Risk for Developing Diabetes: A Systematic Review of Randomized Nutritional Intervention Studies. Can. J. Diabetes.

[126] Jäger R., Kerksick C.M., Campbell B.I., Cribb P.J., Wells S.D., Skwiat T.M., Purpura M., Ziegenfuss T.N., Ferrando A.A., Arent S.M. (2017). International Society of Sports Nutrition Position Stand: Protein and exercise. J. Int. Soc. Sport. Nutr..

[127] Smith A., Gray J. (2016). Considering the benefits of egg consumption for older people at risk of sarcopenia. Br. J. Community Nurs..

[128] Papanikolaou Y., Fulgoni V.L. (2018). Egg Consumption in Infants is Associated with Longer Recumbent Length and Greater Intake of Several Nutrients Essential in Growth and Development. Nutrients.

[129] Rowan H., Brown A. (2023). Infant egg consumption during introduction to solid food remains low in the UK but increases with infant age and a baby led weaning approach. J. Hum. Nutr. Diet..

